# 

**Published:** 1826-08

**Authors:** 


### MONTHLY LIST OF MEDICAL BOOKS.

[*It having been hinted to us that gentlemen, sending copies of their Works, prefer having their titles given at length, it is our intention, in future, to comply with this suggestion: but it is to be observed, that no books can be entered on this List except those sent to us for the purpose;—finding, in the lists hitherto transmitted, that the names of works have frequently been given as published, which have not appeared for weeks, or even months, after.*]

An Elementary System of Physiology. By John Bostock, m.d. f.g.s., &c.—London, 1826.

A Treatise on Diet; with a View to establish, on practical grounds, a System of Rules for the Prevention and Cure of the Diseases incident to a Disordered State of the Digestive Functions. By J. A. Paris, m.d. Fellow of the Royal College of Physicians, &c. &c.—London, 1826.

Remarks on the late Attempt to subvert the Charter of the Royal College of Surgeons; with a dispassionate Examination of some of the Regulations of the Court. To which are subjoined, Animadversions on the evil Tendency of the "Lancet;" and Observations, respectfully addressed to general Practitioners, on the best Means of maintaining their respectability and privileges. By William Cooke, Member of the Royal College of Surgeons, Editor of an Abridgment of "Morgagni de Sed. et Causis," Secretary to the Hunterian Society, &c.

Phrenology, in connexion with the Study of Physiognomy. By G. Spurzheim, m.d. of the Universities of Vienna and Paris, and Licentiate of the Royal College of Physicians of London. Part I. Characters. With thirty-four Plates.—London, 1826.

Observations on the Artificial Mineral Waters of Dr. Struve, of Dresden, prepared at Brighton: with Cases. By W. King, m.d. Fellow of the Royal College of Physicians, London; late Fellow of St. Peter's College, Cambridge.—Brighton, 1826.

Refutation de la Doctrine Medicale de M. le Docteur Broussais, et Nouvelle Analyse des Phenomenes de la Fievrê, par L. Castel, &c. &c.—Paris.

The London Medical and Physical Journal *professedly contains an account of the most important improvements which are made in Medicine and the collateral branches of Science: and it has been thought that, in addition to the means hitherto adopted for conveying this information, considerable advantage would be deduced from giving each month a history of such* Cases *occurring at* Public Institutions, *as might seem calculated to illustrate any points in pathology or practice. On mentioning this idea to the Physicians and Surgeons connected with some of the principal Hospitals and Dispensaries in the metropolis, the utmost readiness was manifested by these gentlemen to have an account of the cases under their care laid before the public; while every facility was promised for obtaining the necessary information. The Editor begs respectfully to express his sense of the liberality which has thus been shown by his professional brethren; and, while he absolves them from all responsibility with regard to the accuracy of the details, he pledges himself to take every means in his power of ascertaining the authenticity of the cases which he publishes: at the same time he requests it to be understood, that he will be ready to correct any misstatement, should such inadvertently be made.*

*The manner in which it appears to the Editor most eligible to arrange these, is according to subjects,—giving in succession a few cases of the same disease, particularly where there is any difference in the symptoms or treatment, the detail of which is likely to prove instructive. It is obvious that this object cannot be accomplished in every instance; but, with a view to secure its fulfilment as frequently as possible, it is intended to make the Reports retrospective, not confining them exclusively to those cases which are of recent occurrence.*

*The Profession in general, and our old Correspondents in particular, are respectfully invited to favour us with Cases, Facts, and information of any kind connected with Medicine and the collateral Sciences.*

### NOTICE TO CORRESPONDENTS.

*Communications have been received from* Dr. Weatherall, Dr. Macandrew, Mr. Hutchinson, Mr. Mackenzie, Mr. Chevalier, Mr. Gullan, Mr. Frost, Mr. Tweedale, Mr. Hamilton, Mr. Fraser, "A Pupil," and Mr. King.

